# Safety evaluation of Bon-santé cleanser® polyherbal in male Wistar rats

**DOI:** 10.1186/s12906-016-1188-8

**Published:** 2016-07-07

**Authors:** O. E. Kale, O. Awodele

**Affiliations:** Department of Pharmacology, Benjamin Carson (Snr.) School of Medicine, Babcock University, Ilisan Remo, Ogun Nigeria; Department of Pharmacology, Therapeutics, and Toxicology, College of Medicine, Idi-Araba Campus, University of Lagos, Lagos, Nigeria

**Keywords:** Polyherbal, Bon-santé cleanser®, Acute oral and intraperitoneal toxicity, Herbotherapy, Safety study

## Abstract

**Background:**

The potential harm of medicinal herbs has been recently observed following herbal toxicity studies after ingestion of polyherbal remedies. This was the rationale for the food and drug regulatory agency decision for thorough safety evaluation of herbal medicines. Androgenic, antipyretic, analgesic and anti-inflammatory potentials as well as chemical compositions of extracts of *massularia acuminata, terminalia ivorensis, anogeissus leiocarpus and macuna pruriens* respectively have been documented*.* Thus*,* Bon-santé cleanser® (BSC) is formulated from these medicinal plants with the intention to boost body hormones and energizes the body. Considering the wide usage of BSC, we investigated on its safety in male Wistar rats.

**Methods:**

Thirty-two male Wistar rats weighing 201.9 ± 7.5 g were grouped into four treatment groups of eight per group. Group I, (control) received distilled water (10 ml/kg). Groups II-IV received 250 mg/kg, 500 mg/kg and 1000 mg/kg of BSC per oral respectively. Each group was treated for sixty days.

**Results:**

Acute toxicity test, in male Wistar albino mice, showed that LD_50_ was 600 mg/kg via *i.p.* while 4 g/kg was nonlethal after oral administration in mice. Hepatic and renal biomarker enzymes were unaltered in all rats. Increased in PCV (*p* <0.05) was observed at 500 mg/kg. BSC modulates antioxidants biomarkers following sub-chronic administration and increased serum Na^+^ (*p* >0.05). BSC at 1000 mg/kg caused mild inflammation of the liver and heart but not kidneys histologically.

**Conclusions:**

BSC has been found to be relatively safe in Wistar rats. Although, our findings indicate that herbal therapy with BSC should be done with caution as a mild alteration in the liver and heart architectures were observed.

## Background

Up to now, Africa still provides a non-negligible contribution to overall complementary and alternative medicines despite the fact that traditional medical practitioners have been using crude drug extracts for several years [1–4]. Thus, there is rationale in investigating herbal toxicity within the African health care system [5, 6], given that a sizable part of our population regularly use them. Since the majority of these products have not been rigorously screened, scientific studies on their uses and toxicological profile will open up new vistas of rational usage of these herbal preparations [5, 7, 8].

Bon-santé cleanser® (BSC) is a marketed polyherbal formula manufactured by Dabiron Natural Life Care in Nigeria. BSC comprises of different constituents as follows: *anogeissus leiocarpus (DC., family Combretaceae), terminalia ivorensis (A. Chev.), massularia acuminata (G.Don,) Bullock ex Hoyle and macuna pruriens (L.,) DC (fabaceae).* formulated into capsule. It is interesting to note that the androgenic, antipyretic, analgesic and anti-inflammatory potentials of the extracts of the aforementioned medicinal plants have been documented [9–16]*.* And recently, Oriola et al. [17] reported a new bioactive thiophenolic glycoside from the leaf of *M. acuminata* in favour of the antibacterial activities [18] and androgenic potentials [19] as well as pro-sexual effects [16]. Ponou et al. [20, 21] demonstrated the present of a dimeric antioxidant and cytotoxic triterpenoid saponins from *T. ivorensis* and have also synthesized a novel 3‐Oxo‐and 3, 24‐Dinor‐2, 4‐secooleanane‐Type triterpenes from this plant. Olugbami et al. [22] using an in vitro study have also unveiled the antioxidant potential, phenolic and flavonoid contents of extract of *A. leiocarpus* while Amadu et al. [23] reported the cytotoxic activity of the same against Ehrlich Ascites Carcinoma cells. Study by Hamzaoui et al. [24] has successfully shown efficient fractionated yields that contained triterpenes, ellagic acid derivatives, flavonoids and phenolic compounds from *A. leiocarpus.* Another recognition strategy based on 13C NMR used by Hubert et al. [25] achieved seven constituents natural metabolites in a crude *A. leiocarpus.* In addition, Josephine and Janardhanan [26] and Perumal et al. [27] have dissected separately the proximate composition, seed protein fractions, amino acid composition, minerals and anti-nutritional factors in *M. pruriens* with high contents of crude protein and crude lipids. Interestingly, aside L-3,4-dihydroxyphenylalanine, *M. pruriens* was found to be rich in minerals such as K, Mg and P. [26, 28].

The androgenic potential of aqueous extract of *massularia acuminata* was reported in male rats [10, 16]. Similarly, Shuklaet al. [11] demonstrated improved male fertility of *macuna pruriens* by its action on the hypothalamus-pituitary-gonadal axis. More so, *anogeissus leiocarpus and T. ivorensis* were explored and verified for their local uses as antipyretics, analgesics and anti-inflammatory effects in rodents [9, 12–14]. *A. leiocarpus* also showed endothelium-independent and endothelium-dependent vasorelaxation action [15]. The pharmacological activities of *M. acuminata*, *T. ivorensis, A. leiocarpus and M. pruriens* have been adduced to be due to the presence of glycosides [16], dimeric antioxidants [20, 21], phenolics [22] and flavones respectively [26, 27]. Considering the wide usage of BSC, coupled with the warning by the National Agency for Food and Drug Administration and Control (NAFDAC) that it has not been evaluated, we investigated on its safety in male Wistar rats.

## Methods

### Chemicals and drugs

The study was carried out in the Department of Pharmacology, University of Lagos, Lagos Nigeria. Bon-santé cleanser® was obtained from Dabiron Natural Life Care, Nigeria. Thiobarbituric acid (TBA), Ellman’s reagent (DTNB) and 1-Chloro-2, 4,- dinitrobenzene (CDBN) were purchased from Sigma Chemical Company (USA). Reduced glutathione (GSH), Metaphosphoric acid and Trichloroacetic acid (TCA) were purchased from J.I. Baker (USA). Bovine serum albumin fraction V (BSA) was purchased from SRL, India. All other chemicals and reagents used were of analytical grade.

### Method of extraction and preparation of the final formulation

BSC was obtained directly from the Dabiron Natural Life Care in Nigeria. It was assigned Batch number 002 and listed with number A7-5321 L by the National Agency for Food and Drug Administration and Control (NAFDAC). BSC contains *Anogeissus leiocarpus (DC., family Combretaceae) Guill & Perr., Terminalia ivorensis (A. Chev., family Combretaceae), Massularia acuminata (G.Don, family Rubiaceae) Bullock ex Hoyle and Macuna pruriens (L., family leguminosae) DC.* in the ratio 4:2:1:1 respectively. The extraction and formulation procedures complied with the regulatory manual of NAFDAC. In this context, a single capsule of BSC (total content 442 mg) was dissolved in distilled water (80 mg/ml) which was administered via oral gavage according to standard toxicological guidelines.

### Animals

Albino rats of the Wistar strain weighing between 150–300 g were purchased from the animal house of the Redeemers University, Ogun State, Nigeria. The rats were housed under controlled conditions in the experimental animal handling facility of the College of Medicine, University of Lagos, Nigeria. The experimental animal room had a 12 h light/12 h dark schedule and maintained at a temperature of 22 ± 3 °C throughout the study. Animals were fed with commercially available rat pelleted diet (Ladoke Akintola Growers Mash, Nigeria) and were allowed access to water *ad libitum* throughout the period of the experiment. The experimental protocols were approved by the Institutional Animal Care and Use Committee, Department of Pharmacology, Therapeutic and Toxicology, College of Medicine, University of Lagos. Animals were certified fit for the experiment by the Institution’s Animal Health Officers before the commencement of the study. Beddings were changed on alternate days and the animals were sacrificed in a humane manner at the end of the experiment by cervical dislocation. The investigation conforms to the Guide for the Care and Use of Laboratory Animals published by the U. S. National Institutes of Health (NIH Publication No. 85-23, revised 1996)” for studies involving experimental animals and the procedures as documented by Kilkenny et al. [29] for reporting animal research.

### Experimental design

#### Acute oral toxicity test

Thirty (30) male Wistar mice (average weight, 20 g) in separate cages were randomly divided into six groups and allowed to acclimatize for two weeks. Graded doses of BSC of 250, 500, 1000, 2000 and 4000 mg/kg were administered to the animals orally and were allowed access to water *ad libitum*. The control group was administered 0.1 ml distilled water orally. The Mice were observed for 24 h post-treatment for mortality, behavioral changes including hyperactivity, attempting circular movement, leaning on hind limbs, increased feeding habit, scratching of lower jaw immediately after treatment and 2 h after treatment, writings, and agitation. All the animals were further monitored for 14 days post administration.

#### Acute intraperitoneal toxicity test

Thirty (30) male mice (average weight, 20 g) placed in separate cages were randomly divided into five groups. Graded doses of BSC of 200, 400, 800 and 1600 mg/kg were administered intraperitoneally. The control group was administered 0.1 ml of distilled water. The Mice were observed 24 h post-treatment for mortality, behavioural changes, and signs of toxicity. At 200 mg/kg (*i.p.*) of BSC, animals showed agitation, climbing, gripping, hyperphagia, leaning on hind limbs and circular movement. Animals that received doses of 400 mg/kg and above showed signs of restlessness, weakness, flattened abdomen, and tears after 2 h of administration. It was observed that at about 6^th^–10^th^ hour, body weight reduced and most animals shivered. Mortality was observed and recorded for each group of animals following 24 h of administration. All animals were further monitored for 14 days post administration. The Median Lethal Dose was estimated according to the method of Finney [30].

#### Subchronic study

Thirty-Two (32) adult male Wistar rats weighing between 150–300 g were divided into 4 groups of 8 rats per group. Group I, the control group, received 10 ml/kg of distilled water daily. The treated animals received only BSC. Groups II, III and IV were administered 250 mg/kg, 500 mg/kg and 1000 mg/kg of BSC respectively. The rats were weighed weekly throughout the course of the experiment. The animals were closely observed for behavioural changes as aforementioned.

### Collection of blood samples and tissues for assays

Sub-chronic administration of BSC in experimental rats was for 60 days. Twenty-four (24) hours following the last administration, blood samples were obtained by ocular puncture into bottles treated with anticoagulants which were either lithium heparin or ethylenediaminetetraacetic acid (EDTA). The animals were sacrificed by cervical dislocation. The anticoagulated blood samples were centrifuged at 4200 rpm for 5 min to separate out the plasma from which all biochemical assays were carried out. The liver, kidney, heart and brain were all harvested, weighed and were homogenized in four volumes of buffer solution (0.1 M, pH 7.4). A portion of each organ was taken out for histological studies. The remaining was weighed and homogenized for biochemical assays.

#### Biochemical assays

##### Liver and renal function tests

Plasma levels of aspartate aminotransferase (AST) and alanine aminotransferase (ALT) were determined following the principle described by Reitman and Frankel [31] while the alkaline phosphates (ALP) was carried out according to the method described by Roy [32] to assess liver function. Renal function was assessed by measuring plasma creatinine (CREA) levels and blood urea nitrogen (BUN) was assayed following the method of Fossati et al. [33] and Skegg’s [34].

##### Proteins and uric acid

In order to assess the synthetic function of the liver, total serum protein (TP) and albumin (ALB) concentrations were determined according to the principles based on the Biuret reaction [35] and bromocresol green reaction [36] respectively. Uric acid (UA) levels were determined according to the methods of Fossati et al. [37].

##### Lipid profiles

Total Serum cholesterol (TC) and Triglyceride (TG) concentrations were estimated following the method described by Trinder [38] by using commercial kits obtained from Randox Laboratories Ltd (Crumlin, UK). High-Density Lipoprotein (HDL) was estimated according to Warnick and Albers [39] while serum low-density lipoprotein (LDL) was calculated using Freidewald formula [40].

##### Antioxidants and oxidative stress

The method of Beutler et al. [41] was followed in estimating the level of reduced glutathione (GSH). Glutathione-S-transferase (GST) activity was determined according to Habig et al. [42]. The level of superoxide dismutase (SOD) activity was determined by the method of Misra and Fridovich [43]. Catalase activity was determined according to the method of Sinha [44]. Lipid peroxidation was determined by measuring the formation of thiobarbituric acid reactive substances (TBARS) according to the method of Varshney and Kale [45].

### Hematological assessments

Packed Cells Volume (PCV), Hemoglobin (Hb), Platelet (PLT), Total White Blood Cell Count (TWBC) were determined using a fully automated haematology analyzer (Pentra-XL 80, Horiba ABX, USA).

### Serum electrolytes

Sodium ion (Na^+^), Potassium ion (K^+^), Chloride ion (Cl^−^), total Calcium ion (tCa^2+^) and intracellular Calcium ion (iCa^2+^) were determined in serum using a Flame Photometer (Sherwood, Model 410).

### Histological assessment

The tissue samples from the liver, kidney and heart for histological examination were passed through the process of fixation, dehydration, clearing, infiltration, embedding, sectioning and staining. To ensure good fixation, the tissues were trimmed to about 5 mm thickness, so as to obtain good fixation. The tissues were then fixed in 10 % formol saline and were then transferred to 50 % alcohol (70 %, 80 %, 85 %, 95 and 100 %), for two hours. Alcohol was removed from the treated tissues by titrating them through first an equal mixture 100 % (absolute) alcohol and xylene for one hour each in that order. Infiltration was carried out twice by passing each tissue through molten paraffin wax in an oven at a temperature of 30 °C for one and a half hours each. The tissues so embedded in molten paraffin wax were later placed on a wooden block and trimmed to size. Serial sections 10μm thick were made using a rotatory microtome. The cut sections were then floated in a warm water bath at a temperature of 30 - 40 °C and were placed slides. Eight sections were obtained from each treated organ from each animal. Four samples were placed on each slide. Microscopic examination was done by using varying magnifications of 10, 40, 100 and 400 to determine if the samples were properly fixed on the slides. Following staining, mounting of sections was carried out using dimethyl paraffinate xylene (DPX) as a mounting agent, after which microscopic examination was done.

### Statistical analysis of data

All data were expressed as mean ± standard error of mean (SEM). Significant differences among the group were determined by one-way analysis of variance (ANOVA) and Post hoc testing was performed for inter-group comparisons for least significant difference (LSD) [46] using the statistical analysis programme for social sciences (SPSS). Graphs were plotted using GraphPad Prism 6. Results were considered to be significant at *p* ≤ 0.05.

## Results

### Acute oral toxicity test

Table 1 shows the results of acute oral toxicity testing of BSC administered at graded doses of 250, 500, 1000, 2000 and 4000 mg/kg respectively. The animals showed no mortality at the doses given. There were also no toxic changes observed with 250 mg/kg while doses of 500 mg/kg and above manifested reduced locomotion, mild scratching of the lower jaw and initial slight hyperactivity followed by weakness in treated mice when compared with control group in first two hours post treatment.

**Table 1:** Effects of acute oral toxicity study after 24 h of administration of Bon-santé cleanser® in mice

Treatments	D/T^K^	Sign of toxicity
Control (Distilled water, 0.1 ml *p.o*)	0/5	No toxic changes observed
BSC (250 mg/kg, *p.o*)	0/5	No toxic changes observed
BSC (500 mg/kg, *p.o*)	0/5	Mild scratching of lower jaw was observed in at least 3 animals in first 2 h
BSC (1000 mg/kg, *p.o*)	0/5	Slight hyperactive, scratching of lower jaw and weakness in at least 3 animals in first 2 h
BSC (2000 mg/kg, *p.o*)	0/5	Slight hyperactive, scratching of lower jaw and weakness in at least 3 animals in first 2 h
BSC (4000 mg/kg, *p.o*)	0/5	Slight hyperactive, scratching of lower jaw and weakness in at least 3 animals in first 2 h

*BSCC* Bon-santé® cleanser capsule, ^*K*^ *D/T* Number of mice death/Total number of mice per group (*n* = 5)

### Acute intraperitoneal toxicity test

Table 2 shows the acute *i.p.* toxicity testing of BSC. Here, we observed mortality or toxic changes in mice administered with BSC at doses of 200, 400, 800 and 1600 mg/kg respectively. At 200 mg/kg (*i.p.*) of BSC, animals showed signs of agitation, climbing, gripping, hyperphagia, leaning on hind limbs, and circular movements. Doses of 400 mg/kg and above showed signs of restlessness, weakness, flattened the abdomen, tears, and increased shallow breathing after 2 h of administration. And at about 6^th^–10^th^ hour, most animals shivered. We obtained a mean lethal dose (LD_50_) of 600 mg/kg.

**Table 2:** Effects of acute intraperitoneal toxicity study after 24 h of administration of Bon-santé cleanser® in mice

Treatments	D/T^K^	Sign of toxicity
Control (Distilled water, 0.1 ml *i.p*)	0/6	No toxic changes observed
BSC (200 mg/kg, *i.p*)	0/6	No toxic changes observed
BSC (400 mg/kg, *i.p*)	2/6	Slight weakness observed in at least 3 animals in first 2 h.
BSC (800 mg/kg, *i.p*)	6/6	Slight weakness observed in at least 3 animals in first 2 h.
BSC (1600 mg/kg, *i.p*)	6/6	Slight weakness observed in at least 3 animals in first 2 h.

*BSC* Bon-santé cleanser®, ^*K*^ *D/T* Number of mice death/Total number of mice per group (*n* = 6). Acute Lethal Dose (LD_50_) = 600 mg/kg

### Liver function

Table 3 shows that the levels of serum alanine aminotransferase (ALT), aspartate aminotransferase (AST) and alkaline phosphatase (ALP) did not change significantly (*p* >0.05) when compared with the control group. BSC at lowest dose (250 mg/kg) reduced ALT and ALP levels respectively. Similarly, there was statistically insignificant (*p* >0.05) dose dependent increase in AST levels when administration BSC was administered at 250 mg/kg (32.4 %), 500 mg/kg (39.2 %) and 1000 mg/kg (51.4 %). ALT levels were insignificantly (*p* >0.05) elevated at doses of 500 mg/kg (41.4 %) and 1000 mg/kg (29.3 %) respectively.

**Table 3:** Effects of subchronic administration of Bon-santé cleanser® on liver function in male rats

Treatments	ALT (Units/L)	AST (Units/L)	ALP (Units/L)
Control (DW, 10 ml/kg)	20.50 ± 3.95	18.50 ± 0.95	38.00 ± 2.94
BSC (250 mg/kg)	18.50 ± 2.99	24.50 ± 5.56	37.50 ± 2.33
BSC (500 mg/kg)	29.00 ± 4.04	25.75 ± 3.88	35.50 ± 5.44
BSC (1000 mg/kg)	26.50 ± 3.10	25.00 ± 3.70	40.25 ± 2.25

Note: Result expressed as mean ± SEM

*BSC* Bon-santé cleanser®, *DW* Distilled water (10 ml/kg), *ALT* Alanine Aminotransferase, *AST* Aspartate Aminotransferase, *ALP* Alkaline Phosphatase

### Lipid profiles

BSC shows a slight reduction (*p* >0.05) in serum TG levels by 11.6 %, 9.8 % and 1.5 % respectively, although, the lowest dose of BSC (250 mg/kg) slightly elevated serum TC levels (Table 4). Serum low density lipoprotein (LDL) was insignificantly (*p* >0.05) lowered by 14.7 % and 4.6 % at doses of 250 mg/kg and 500 mg/kg. BSC (1000 mg/kg) slightly elevated LDL by 28.5 % (*p* >0.05).

**Table 4:** Effects of subchronic administration of Bon-santé cleanser® on serum lipids levels in male rats

Treatments	TC (mg/dl)	TG (mg/dl)	HDL (mg/dl)	LDL (mg/dl)
Control (DW)	91.00 ± 8.19	120.50 ± 9.50	27.00 ± 1.58	58.70 ± 8.90
BSC (250 mg/kg)	99.25 ± 9.25	106.50 ± 8.73	27.75 ± 3.90	50.05 ± 9.38
BSC (500 mg/kg)	117.00 ± 9.25	108.67 ± 16.22	25.50 ± 1.32	56.00 ± 7.40
BSC (1000 mg/kg)	112.50 ± 7.54	118.67 ± 17.46	27.50 ± 1.76	75.40 ± 6.60

Note: Result expressed as mean ± SEM

*BSC* Bon-santé cleanser®, *DW* Distilled water (10 ml/kg), *TC* Total Cholesterol, *TG* Triglyceride, *HDL* High Density Lipoprotein, *LDL* Low Density Lipoprotein

### Renal function, protein synthesis and uric acid

Blood Urea Nitrogen levels was increased by 11.8 %(*p* >0.05) and 13.7 %(*p* >0.05) at 500 mg/kg and 1000 mg/kg respectively as shown in Table 5. BSC (250 mg/kg) also elevated Creatinine levels by 7.1 % (*p* >0.05), but, did not significant alter serum albumin (ALB), total protein (TP) and UA respectively in all subjects. But, BSC doses of 500 mg/kg and 1000 mg/kg, produced insignificantly (*p* >0.05) elevated levels of ALB, TP, and UA in the treated rats.

**Table 5:** Effects of subchronic administration of Bon-santé cleanser® on renal function, serum albumin, total protein and uric acid levels in male rats

Treatments	Control	BSC	BSC	BSC
	(DW)	(250 mg/kg)	(500 mg/kg)	(1000 mg/kg)
UREA (U/L)	9.35 ± 1.74	8.88 ± 1.95	10.43 ± 2.68	10.63 ± 1.33
CREATININE (U/L)	0.28 ± 0.05	0.30 ± 0.04	0.28 ± 0.03	0.28 ± 0.03
ALB (mg/dl)	4.43 ± 0.39	4.05 ± 0.31	5.10 ± 0.29	4.55 ± 0.24
TP (mg/dl)	8.98 ± 0.74	8.73 ± 0.63	9.43 ± 0.77	9.55 ± 0.25
UA (mg/dl)	9.35 ± 1.74	8.88 ± 1.95	10.43 ± 2.68	10.63 ± 1.33

Note: Result expressed as mean ± SEM

*BSC* Bon-santé cleanser®, *ALB* Albumin, *TP* Total Protein, *UA* Uric Acid

### Haematological assessments

BSC shows elevated packed cell volumes at 250 mg/kg (*p* >0.05) and 500 mg/kg (*p* <0.05) respectively (Table 6). Similarly, heamoglobin levels were insignificantly (*p* >0.05) when compared with control as well. BSC increased TWBC at 250 mg/kg by 62.6 % (*p* <0.05) and platelets count by 50.6 % (*p* >0.05) when administered at 500 mg/kg.

**Table 6:** Effects of subchronic administration of Bon-santé cleanser® on hematological parameters in male rats

Treatments	Control	BSC	BSC	BSC
	(DW)	(250 mg/kg)	(500 mg/kg)	(1000 mg/kg)
PCV	31.8 ± 2.7	35.8 ± 1.7	40.0 ± 1.2^*^	26.5 ± 4.1
Hb	10.8 ± 1.0	12.5 ± 0.5	13.4 ± 0.4	10.0 ± 1.2
PLT (x10^9^/L)	293.8 ± 67.2	235.0 ± 48.6	442.5 ± 29.5	171.3 ± 21.1
TWBC (x10^9^/L)	4.8 ± 1.2	7.8 ± 1.4	3.6 ± 0.6	3.9 ± 0.3

Note: Result expressed as mean ± SEM

*BSC* Bon-santé cleanser®, *DW* Distilled water (10 ml/kg), *PCV%* Packed Cell Volume, *Hb* Hemoglobin, *PLTx10* ^*9*^ Platelet, *TWBC* Total White Blood Cell Count

^*^ *p* <0.05 when compared with control group

### Serum electrolytes

BSC causes a dose related insignificantly increased Na^+^ following oral administration from 250 mg/kg through 1000 mg/kg (Table 7). iCa^2+^ levels was decreased at 250 mg/kg(*p* >0.05) while it increased(*p* >0.05) at the middle and highest doses respectively. The serum K^+^, Cl^−^ and tCa^2+^ levels were unaltered in animals.

**Table 7:** Effects of subchronic administration of bon-santé cleanser® on serum electrolytes in male rats

TREATMENTS	Control	BSC	BSC	BSC
	(DW)	(250 mg/kg)	(500 mg/kg)	(1000 mg/kg)
Na^+^	16 .8 ± 1.3	23.9 ± 5.0	25.5 ± 5.6	23.9 ± 3.8
K^+^	135.1 ± 0.9	143.2 ± 18.4	128.9 ± 8.0	126.5 ± 3.8
Cl^−^	98.8 ± 0.8	94.2 ± 0.9	96.2 ± 1.6	96.9 ± 1.2
tCa^2+^	2.41 ± 0.0	2.21 ± 0.1	2.34 ± 0.1	2.32 ± 0.1
iCa^2+^	1.24 ± 0.02	1.08 ± 0.02^*^	1.17 ± 0.04	1.19 ± 0.04

Note: Result expressed as mean ± SEM. b: mmol/L

*BSC* Bon-santé cleanser®, *DW* Distilled water (10 ml/kg), *Na* ^*+*^ Sodium ion, *K* ^*+*^ Potassium ion, *Cl* ^*−*^ Chloride ion, *tCa* ^*+*^ total Calcium ion, *iCa* ^*2+*^ intracellular Calcium ion

^*^ *p* <0.05 when compared with control group

### Tissue proteins

**Fig. 1:**
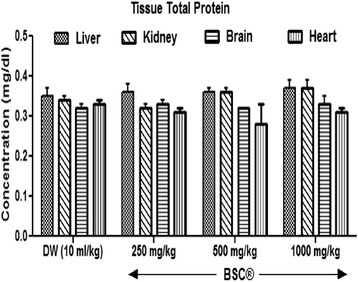
Effects of Bon-santé cleanser® (BSC) on tissue total protein in normal male rats. Result expressed as mean ± SEM; Control: (DW, Distilled water)

Figure 1, BSC produces an increased in the levels of hepatic TP, although, insignificantly (*p* >0.05). No difference was observed in renal, brain and heart TP levels respectively in all subjects when compared with control group (*p* >0.05).

### Lipid peroxidation

**Fig. 2:**
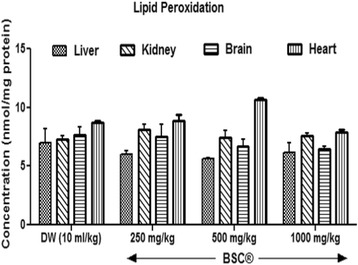
Effects of Bon-santé cleanser® (BSC) on lipid peroxidation in normal male rats. Result expressed as mean ± SEM. DW: Distilled water (10 ml/kg). LPO: Lipid Peroxidation (ie Malondialdehyde)

BSC at doses of 250 mg/kg, 500 mg/kg and 1000 mg/kg shows reduced (*P* >0.05) hepatic and brain LPO (Malondialdehyde, MDA) (Fig. 2). Similarly, low (250 mg/kg) and highest (1000 mg/kg) doses also reduced renal MDA (*P* >0.05). BSC (500 mg/kg) shows slight elevation (*P* >0.05) in MDA levels. Both 250 mg/kg and 500 mg/kg doses elevated MDA levels in heart, although, insignificantly when compared with control (*p* >0.05).

### Tissue anti-oxidants

#### Hepatic antioxidants

**Fig. 3:**
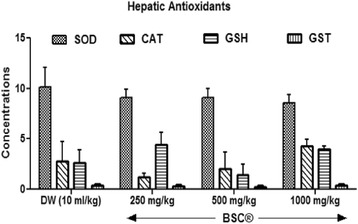
Effects of Bon-santé cleanser® (BSC) on hepatic antioxidants in normal male rats. Result expressed as mean ± SEM. DW: Distilled water (10 ml/kg); GSH: Reduced Glutathione (μmol/mg protein); CAT: Catalase (μmol/ml/mg protein); SOD: Superoxide Dismutase Activity (μmol/min/mg protein); GST: Glutathione-S-Transferase (μmol/ml/min)

BSC shows at low (250 mg/kg) and highest (1000 mg/kg) doses an increased (*p* <0.05) hepatic GSH levels by 68 % and 49.2 % respectively (Fig. 3). No observable statistical difference in GST, superoxide dismutase and the control. But, a 1000 mg/kg of BSC indicated elevated hepatic CAT levels by 54.1 % (*p* <0.05).

#### Renal antioxidants

**Fig. 4:**
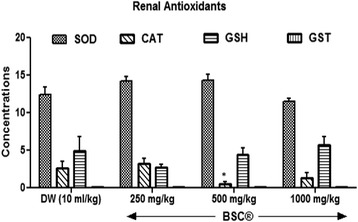
Effects of Bon-santé cleanser® (BSC) on renal antioxidants in normal male rats. Result expressed as mean ± SEM. ^*^ *p* <0.05 when compared with control group. DW: Distilled water (10 ml/kg); GSH: Reduced Glutathione (μmol/mg protein); CAT: Catalase (μmol/ml/mg protein); SOD: Superoxide Dismutase Activity (μmol/min/mg protein); GST: Glutathione-S-Transferase (μmol/ml/min)

There were increased GSH levels in the kidneys at highest BSC dose (1000 mg/kg) used in this present study (Fig. 4). However, renal GST levels decreased given all administered doses of BSC. Only the lowest dose (250 mg/kg) shows increased CAT levels. On the other, both 250 mg/kg and 500 mg/kg elevated renal SOD levels but insignificant (*p* >0.05).

#### Heart antioxidants

**Fig. 5:**
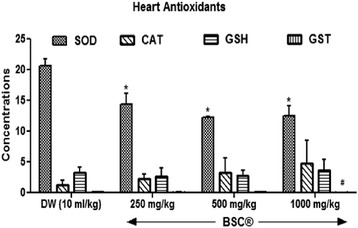
Effects of Bon-santé cleanser® (BSC) on heart antioxidants in normal male rats. Result expressed as mean ± SEM. ^#^ *p* <0.05 and ^*^ *p* <0.001 when compared with control group. DW: Distilled water (10 ml/kg); GSH: Reduced Glutathione (μmol/mg protein); CAT: Catalase (μmol/ml/mg protein); SOD: Superoxide Dismutase Activity (μmol/min/mg protein); GST: Glutathione-S-Transferase (μmol/ml/min)

BSC highest dose (1000 mg/kg) produces elevated heart GSH levels by 9.7 % and decreases (*p* <0.05) heart GST levels in treated rats by 54 % (Fig. 5). In addition, CAT levels increased in the heart at 500 mg/kg and 1000 mg/kg (*p* >0.05). There were decreased (*p* <0.05) SOD levels in the heart of treated rats, in a dose related manner by 30.1 % (250 mg/kg), 40.5 % (500 mg/kg) and 39.1 % (1000 mg/kg) respectively.

#### Brain antioxidants

**Fig. 6:**
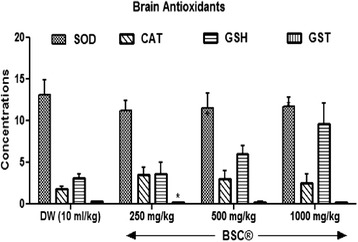
Effects of Bon-santé cleanser® (BSC) on brain antioxidants in normal male rats. Result expressed as mean ± SEM. ^*^ *p* <0.05 when compared with control group. DW: Distilled water (10 ml/kg); GSH: Reduced Glutathione (μmol/mg protein); CAT: Catalase (μmol/ml/mg protein); SOD: Superoxide Dismutase Activity (μmol/min/mg protein); GST: Glutathione-S-Transferase (μmol/ml/min)

An administration of BSC at doses used in this study produces a dose dependent increased (*p* <0.05) in brain GSH levels by 18.8 %, 96.4 % and 212.3 % respectively (Fig. 6). More so, BSC at 250 mg/kg shows a decreased brain GST levels in the treated animals (*p* <0.05). Brain CAT levels was also elevated (*p* <0.05) given 500 mg/kg and 1000 mg/kg respectively. However, there was a decrease (*p* >0.05) in brain SOD levels in the treated rats.

### Body and organ system weights

**Fig. 7:**
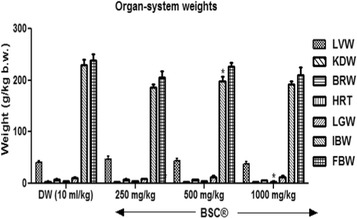
Effects of Bon-santé cleanser® (BSC) on organ system body weight in normal male rats. Result expressed as mean ± SEM. ^*^ *p* <0.05 when compared with control group. DW: Distilled water (10 ml/kg); LVW: Liver weight; KDW: Kidney weight; BRW: Brain weight; HW: Heart weight; LGW: Lungs weight

The effects of sub-chronic administration of BSC on body weights in male rats administered with 250 mg/kg and 1000 mg/kg were not significantly (*p* >0.05) different from those of control groups (Fig. 7). However, only 500 mg/kg of BSC ably increased (*p* <0.05) body weights. And similarly, a 1000 mg/kg reduced (*p* <0.05) heart as well.

### Histological assessments

#### Control group (Distilled Water, 10 ml/kg)

**Fig. 8:**
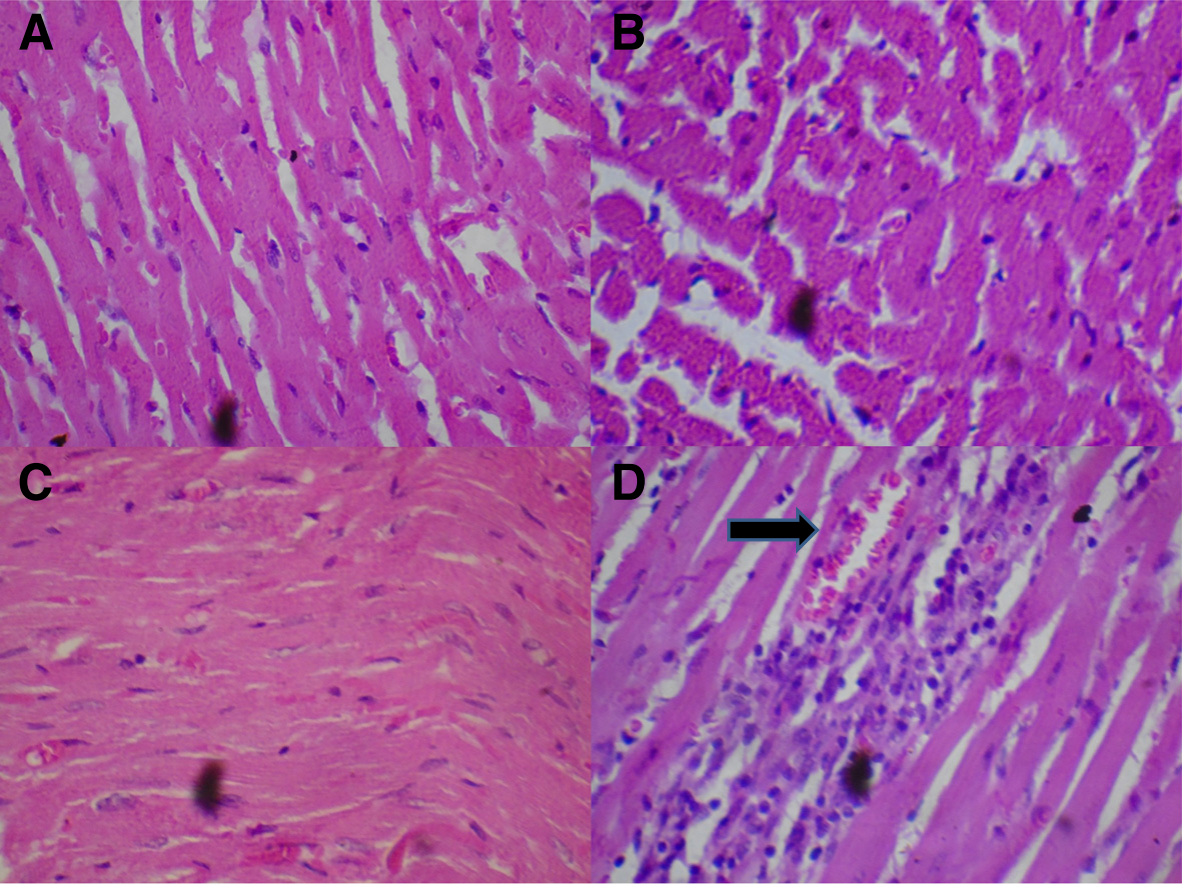
Control (Distilled Water, 10ml/kg) shows normal heart muscle (**a**). BSC (250mg/kg) shows normal heart (**b**). BSC (500mg/kg) shows normal heart (**c**). BSC (1000mg/kg) shows dense aggregates inflammatory heart (**d**) (see *arrow*). (H & E stain, Mag. X400). BSC= Bon-santé cleanser^®^

Figure 8: Control (Distilled Water, 10 ml/kg, A) and BSC (250 mg/kg, B; 500 mg/kg, C) showed normal heart. However, BSC (1000 mg/kg, D) shows dense aggregates inflammatory heart (see arrow). (H & E stain, Mag. X400). (BSC = Bon-santé cleanser®).

#### BSC (250 mg/kg)

**Fig. 9:**
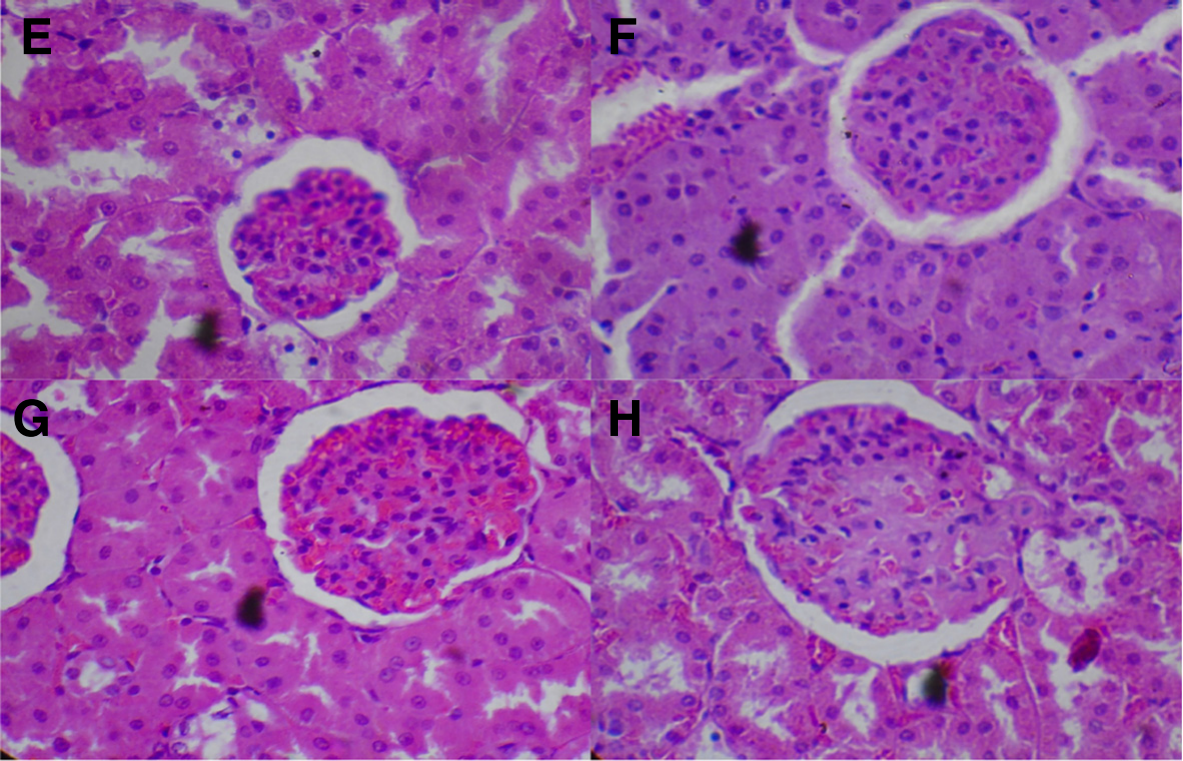
Control (Distilled Water, 10ml/kg) shows normal kidney (**e**). BSC (250mg/kg) shows normal kidney (**f**). BSC (500mg/kg) shows normal kidney (**g**). BSC (1000mg/kg) shows normal kidney (**h**) (H & E stain, Mag. X400). (BSC= Bon-santé cleanser®)

Figure 9: Control (Distilled Water, 10 ml/kg, E) and BSC (250 mg/kg, F; 500 mg/kg, G; 1000 mg/kg, H) showed normal kidney (H & E stain, Mag. X400). (BSC = Bon-santé cleanser®).

#### BSC (500 mg/kg)

**Fig. 10:**
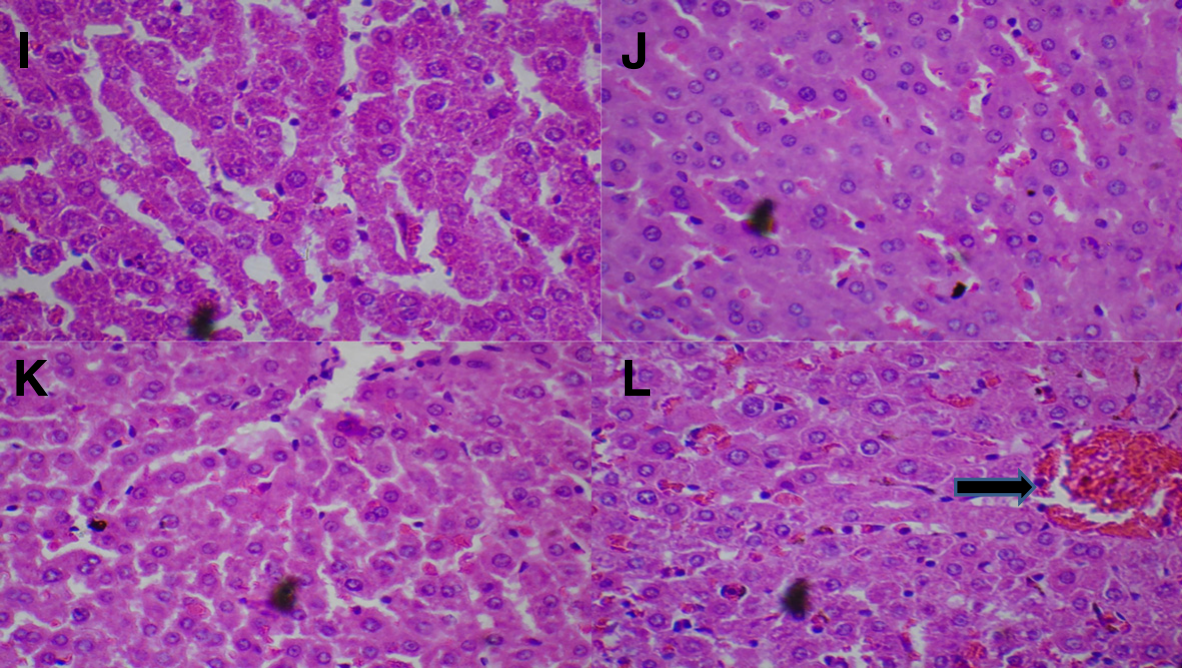
Control (Distilled Water, 10ml/kg) shows normal liver (**i**). BSC (250mg/kg) shows normal liver (**j**). BSC (500mg/kg) shows normal liver (**k**). BSC (1000mg/kg) shows congestion of the hepatic sinusoids and the central vein liver (**l**) (see *arrow*). (H & E stain, Mag. X400). (BSC = Bon-santé cleanser®)

Figure 10: Control (Distilled Water, 10 ml/kg, I). BSC (250 mg/kg, J; 500 mg/kg, K) showed normal liver. But, BSC (1000 mg/kg, L) shows congestion of the hepatic sinusoids and the central vein (see arrow). (H & E stain, Mag. X400). (BSC = Bon-santé cleanser®).

## Discussions

There is a new global interest in the use of alternative means of maintaining and healing sick people [47]. The WHO fact sheet (No.134) estimates that about 80 % of the populations in African and Asian countries rely on traditional medicine for their primary healthcare [48]. It also recognizes traditional medicine as ‘an accessible, affordable and culturally acceptable form of healthcare trusted by large numbers of people, which stands out as a way of coping with the relentless rise of diseases in the midst of soaring health-care costs and nearly universal austerity [47]. There are many reasons for this new phenomenon. The first is that many of the producers describe multifaceted great healing properties to these alternative means [5, 6, 49]. The second is that they propose that these therapies are natural and so have less or little side effects [5, 50]. Finally because of poverty and the inability to pay for some of the western medicines that have verifiable efficacies many patients turn to herbal medicines. Unfortunately, there are evidence to show that the majority of these herbal medicines have not been as well screened for efficacy and toxicity when compared to their western counterparts [1, 51]. As toxicity studies and scientific investigations into these herbal remedies are often expensive [52], it is now left to the scientific community to investigate some of the claims of the manufacturers of these herbal preparations to verify or refute their claims. This is an important public health service that must not be overlooked if it is really true that academics must contribute to safeguarding the health of our people.

In view of the foregoing, scientific investigations of alternative means to healthcare are now focusing on safety and toxicological evaluation of herbal formulation [53–55]. Our group decided to study BSC for the following reasons. BSC contains *A. lerocarroa, T. accularia, M. accuminata and M. pruiens* formulated into a capsule. Several authors at different times have contributed to the elucidation of the chemical compositions of the individual component of the popular BSC. Also, The chemical compositions and hypotheses on folklore medicine uses of these plants have been well documented [17–21, 23–28, 56]. However, some investigators have shown that there is potential to augment the individual toxicities in herbal preparations when mixed [5, 6, 8, 55, 57–59]. To the best of our knowledge, not much is known in the literature with respect to the toxicity of the components of the herbs contained in the BSC capsule. Thus, there is rational in carrying out toxicological studies on this herbal preparation as it is on the open market, and the manufacturers claim they have many clients. Therefore, we investigated the Safety evaluation of BSC when ingested orally in male Wistar rats.

The determination of acute toxicity testing is usually a step *ab inicio* in the assessment and evaluation of the toxic characteristics of a substance [60]. Our results show that when BSC is given orally, the herbal preparation was relatively safe as there was no mortality during the acute toxicity tests. This is an important finding as the major route of administration of herbal medicines is oral. The acute oral and intraperitoneal LD_50_ in mice were >4000 mg/kg and 600 mg/kg respectively.

Nevertheless, there were small but statistically insignificant changes in serum liver and renal enzymes as well as serum electrolytes (Tables 3, 5 and 7). Whether this will later translate into outright hepatic and renal damage with the chronic administration is very hard to tell. One important outcome of this study is the increase in PCV (Table 6) and decrease in LDL (Table 4) with oral administration. While these results may look exciting, it is too early to determine whether BSC could be deployed as a lipid modulating remedy either on its own or as an adjunct to well-known compounds that carry out this role in the health system. No histological changes following a subchronic oral administration were seen on the kidneys unlike mild to moderate alterations that were observed in the liver and heart when the highest dose (1000 mg/kg) was administered (Figs. 8, 9 and 10). However, the limitation of our study lies in the duration since a chronic toxicity evaluation of BSC would be necessary in order to comment on the long-term outcomes.

Since there have been suggestions that botanicals, when used alone or in combination with other medications, could precipitate severe organ system toxicity [61]. Our observation is that treatment with different doses of BSC did not result in predictable changes with graded doses (Tables 3, 4, 5, 6, and 7 & Figs. 1, 2, 3, 4, 5, and 6). Thus, there is a lot of cautions to be expressed in humans as our study indicate altered levels of hepatic biomarkers and some antioxidants, though, it does not pinpoint the part of departure between dose and biochemical outcomes. More so, there was a small improvement in lipid profile at low doses. Thus, at this stage, it could be suggested that further observations be made in humans who have consumed the medicine in the past to verify its usefulness and comments on side effects.

## Conclusion

BSC has been found to be relatively safe in Wistar rats. Although, our findings indicate that herbal therapy with BSC should be done with caution as a mild alteration in the liver and heart architectures were observed.

## Abbreviations

BSC, Bon-santé cleanser®; ED_50_, Mean Effective Dose; LD_50_, Mean Lethal Dose
